# Supplementary material for: Adverse Events Comparison of Double Beta-Lactam Combinations for Bloodstream Infections: Ampicillin plus Ceftriaxone and Ampicillin/Cloxacillin
Source: Antibiotics (Basel). 2024 Jul 25;13(8):696. doi: 10.3390/antibiotics13080696 (PMC11350733; doi:10.3390/antibiotics13080696)
Supplement: Supplementary file 1 [file antibiotics-13-00696-s001.zip › antibiotics-3097227-supplementary.pdf]

Supplemenatary Materials

Table S1. baseline characteristics of patients with bacteremia after propensity score matching.

n	ABPC(21)	ABPC/MCIPC (21)	Standardized Mean Difference	p
age (SD)	68.4(17.5)	64.1(17.1)	0.248	0.426
male(%)	9(42.9)	6(28.6)	0.149	0.334
congestive heart failure(%)	4(19.0)	8(38.1)	0.211	0.172
diabetes(%)	7(33.3)	5(23.8)	0.105	0.495
respiratory disease(%)	10(47.6)	10(47.6)	0.000	1.000
myocadiac infarction(%)	0(0.0)	1(4.8)	0.156	1.000
collagen disease(%)	1(4.8)	0(0.0)	0.156	1.000
liver dysfunction(%)	2(9.5)	3(14.3)	0.074	1.000
cancer(%)	5(23.8)	3(14.3)	0.121	0.697
chronic kidney disease(%)	8(38.1)	5(23.8)	0.155	0.317
cerebrovascular(%)	4(19.0)	4(19.0)	0.000	1.000
hypertension(%)	14(66.7)	12(57.1)	0.098	0.525
HIV(%)	0(0.0)	0(0.0)		
qSofa>=2(%)	5(23.8)	7(33.3)	0.105	0.495
CLDM(%)	5(23.8)	7(33.3)	0.105	0.495
VCM(%)	5(23.8)	5(23.8)	0.000	1.000
AG(%)	1(4.8)	3(14.3)	0.162	0.606
L-AMPHB(%)	0(0.0)	0(0.0)		
ACV(%)	0(0)	0(0)		
NSAIDS(%)	4(19.0)	5(23.8)	0.058	1.000
ACE inhibitor/ARB(%)	6(28.6)	7(33.3)	0.052	0.739
Diuretics(%)	3(14.3)	6(28.6)	0.174	0.454
Chemotherapy(%)	0(0.0)	0(0.0)		
Calcineurin Inhibitor(%)	0(0.0)	0(0.0)		
Contrast CT(%)	9(42.9)	10(47.6)	0.048	0.757
On admission eGFR(SD)	100.5(70.6)	85.5(34.1)	0.270	0.387
T-bil(SD)	0.79(0.55)	0.75(0.58)	0.067	0.828
ALP(SD)	287(123)	277(135)	0.073	0.814
AST(SD)	34.4(14.5)	46.1(66.6)	0.241	0.443
ALT(SD)	31.3(15.3)	34.6(51.5)	0.087	0.781
GTP(SD)	63.8(68.1)	51.9(43.0)	0.209	0.502
CRP(SD)	14.1(12.3)	14.2(10.6)	0.008	0.980
WBC(SD)	10.0(6.09)	11.2(5.29)	0.197	0.527
Hgb(SD)	11.1(2.21)	11.8(3.49)	0.240	0.442
PLT(SD)	223(143)	216(134)	0.045	0.885
duration of treatment(SD), day	15.7(13.7)	21.1(16.2)	0.365	0.244
Use of mechanical ventilation(%)	2(9.5)	3(14.3)	0.074	1.000
use of vasopressor(%)	3(14.3)	4(19.0)	0.064	1.000

Variables adjusted in the study with caliper value=0.2; Abbreviation: ABPC, ampicillin; ABPC/MCIPC, ampicillin/cloxacillin; SD, standard deviation; HIV, human immunodeficiency virus; CLDM, clindamycin; VCM, vancomycin; AG, aminoglycoside; L-AMPHB, liposomal-amphotericin B; ACV, acyclovir; NSAIDS, Non-Steroidal Anti-Inflammatory Drugs; ACE, Angiotensin-Converting Enzyme; ARB, Angiotensin II Receptor Blocker; CT, computed tomography; eGFR, estimated

glomerofiluter rate; ALP, alkaline phosphatase; AST, Aspartate Aminotransferase; ALT, Alanine Aminotransferase; GTP, Guanosine Triphosphate; CRP, C-related protein; WBC, white blood cell count; Hgb, hemoglobin; PLT, platelate.

Table S2. patietnt's outcome after propensity score matching.

n	ABPC(21)	ABPC/MPIPC(21)	*p*
duration of staying hospital(SD)	34.7(39.4)	41.8(16.4)	0.451
**AKI KDIGO Grade1-3(%)**	**0(0.0)**	**5(23.8)**	**0.048**
Grade2-4 leukopenia(%)	0(0.0)	0(0.0)	
Grade2-4 anemia(%)	9(42.9)	10(47.6)	0.757
Grade2-4 thrombocytopenia(%)	1(4.8)	4(19.0)	0.343
Grade2-4 T-bil elevation(%)	1(4.8)	1(4.8)	1.000
Grade2-4 ALP elevation(%)	1(4.8)	1(4.8)	1.000
Grade2-4 G-GTP elevation(%)	8(38.1)	4(19.0)	0.172
Grade2-4 AST elevation(%)	2(9.5)	4(19.0)	0.663
Grade2-4 ALT elevation(%)	2(9.5)	3(14.3)	1.000
30-day mortality(%)	0(0.0)	1(4.8)	1.000
90-day mortality(%)	0(0.0)	1(4.8)	1.000
30-day ICU admission(%)	0(0.0)	0(0.0)	

Abbreviation: ABPC, ampicillin; ABPC/MCIPC, ampicillin/cloxacillin; SD, standard deviation; AKI, acute kidney injury; ALP, alkaline phosphatase; AST, Aspartate Aminotransferase; ALT, Alanine Aminotransferase; GTP, Guanosine Triphosphate; ICU, intensive care unit.

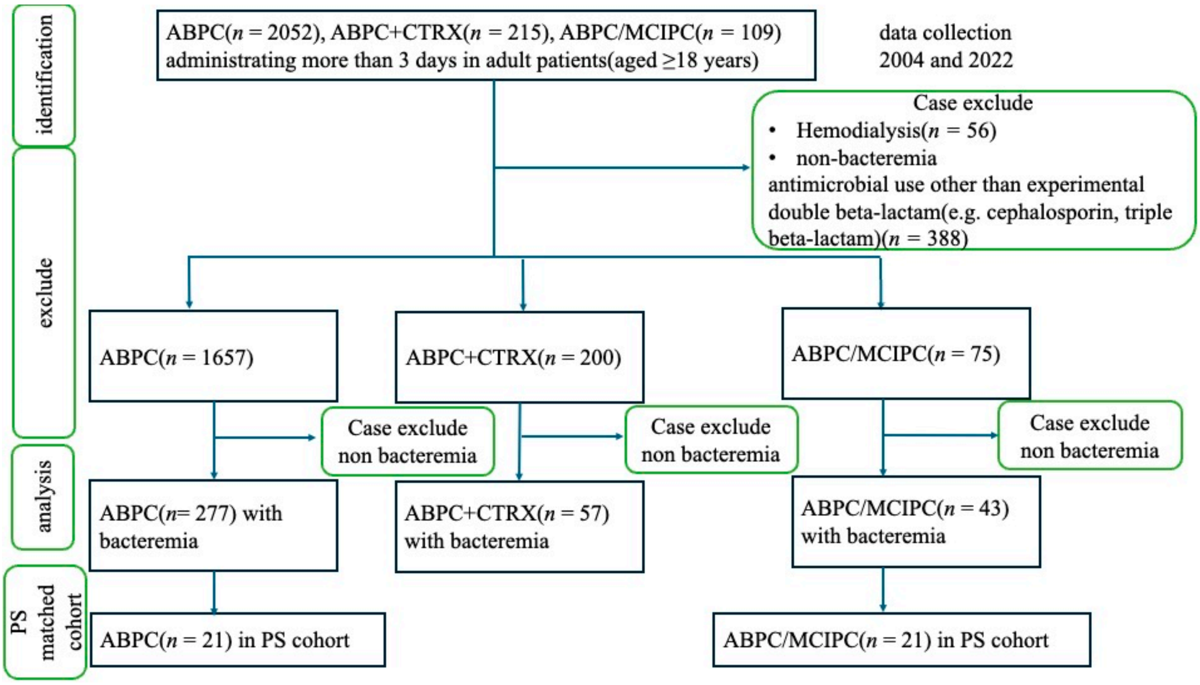

Figure S1. Selection flowchart for episodes of ABPC, ABPC+CTRX, and ABPC/MPIPC with bacteremia. ABPC, ampicillin; CTRX, ceftriaxone; MCIPC, cloxacillin; PS, propensity score.
